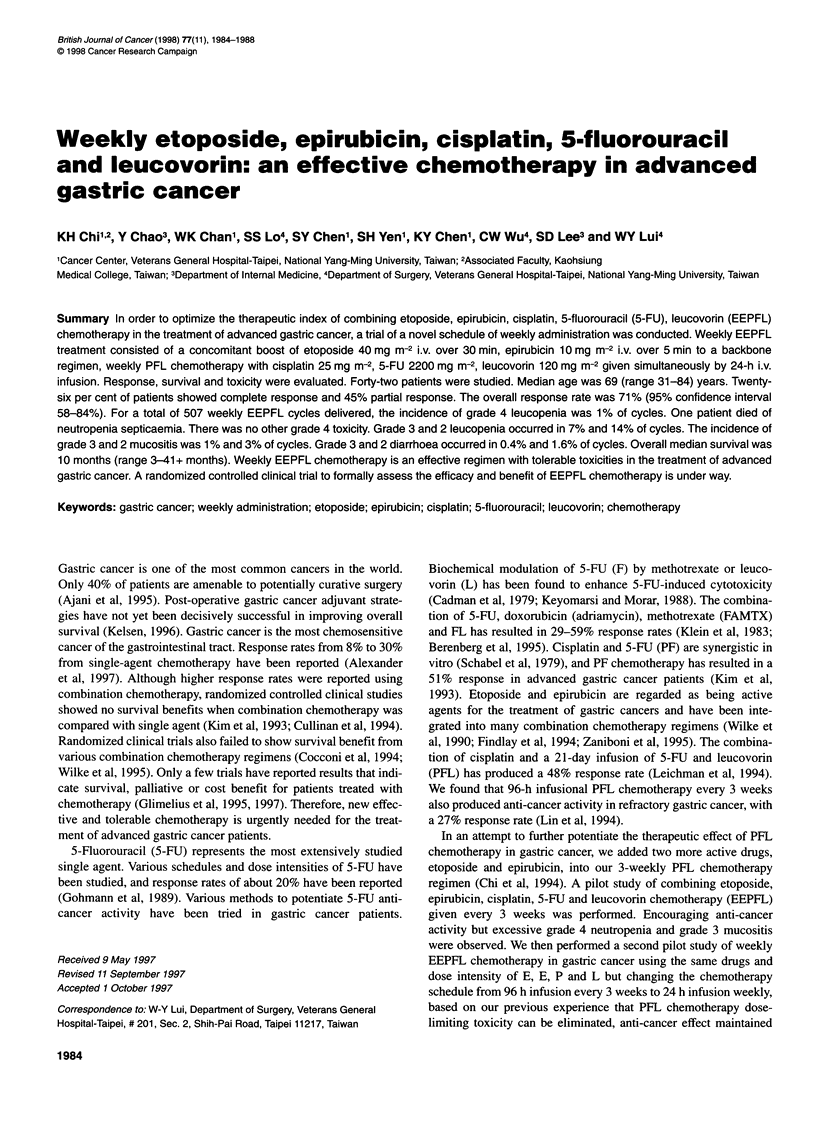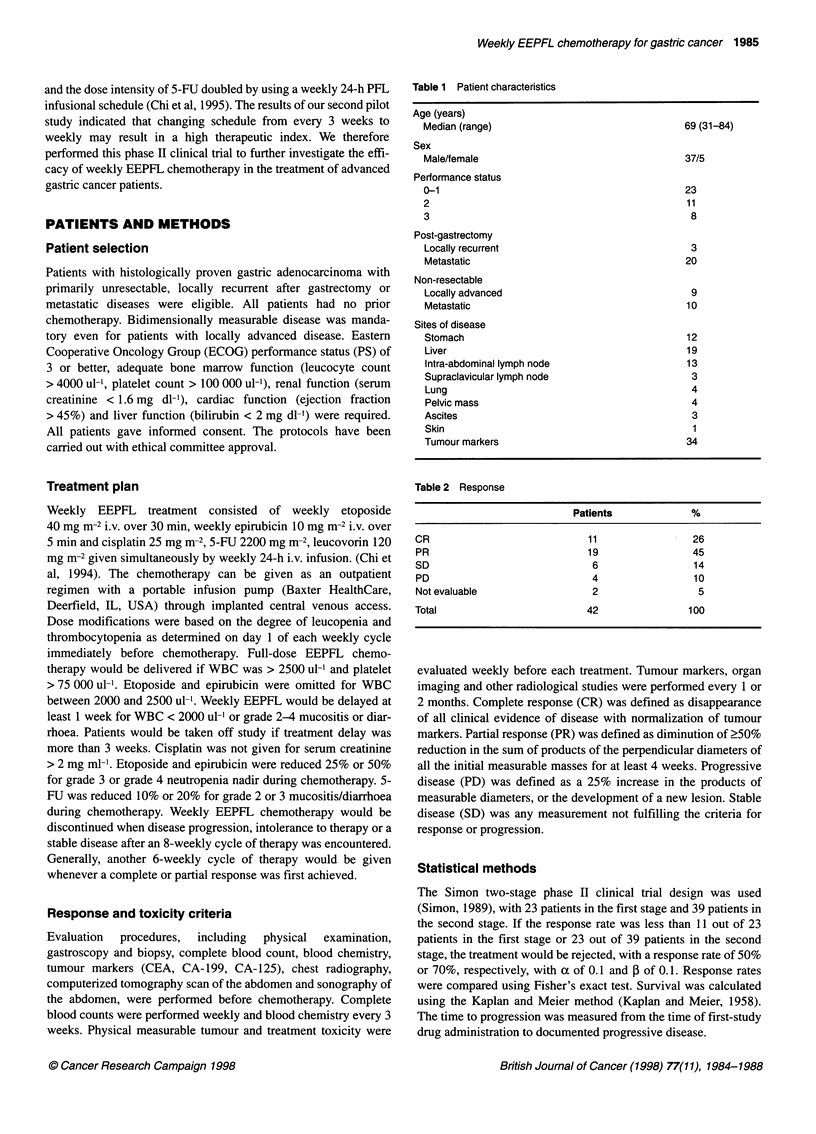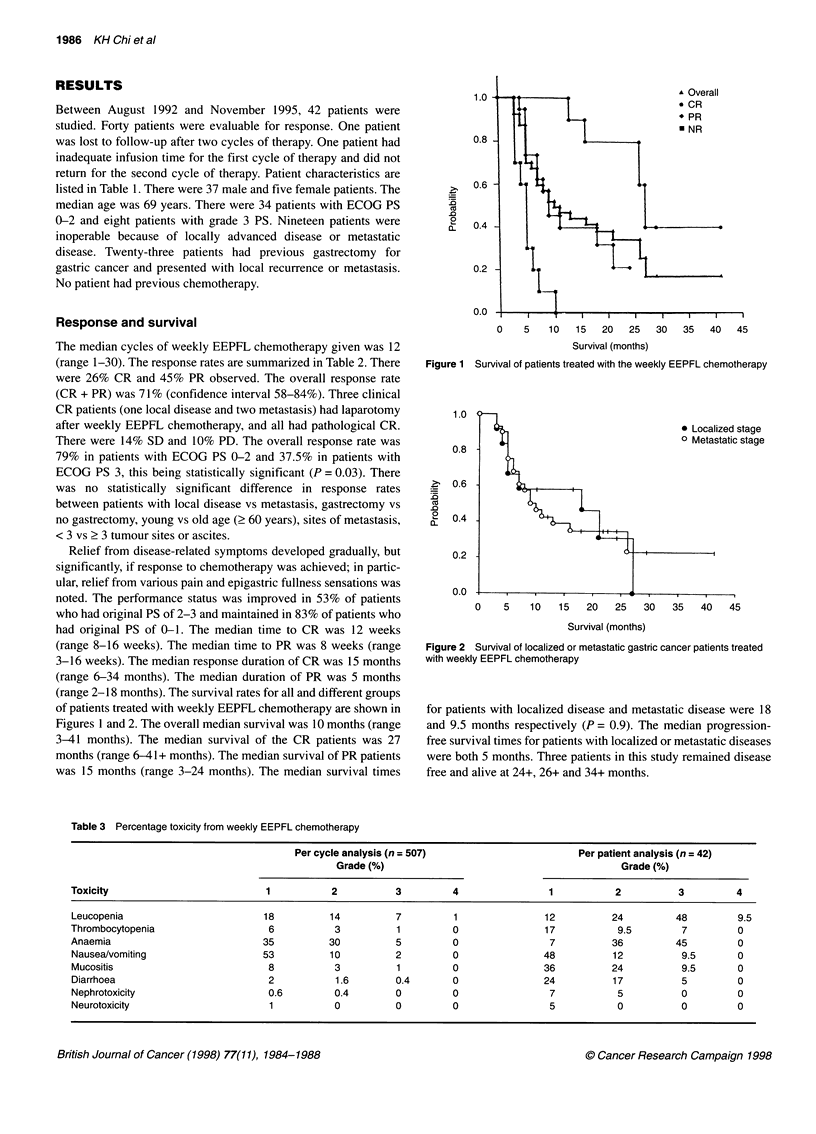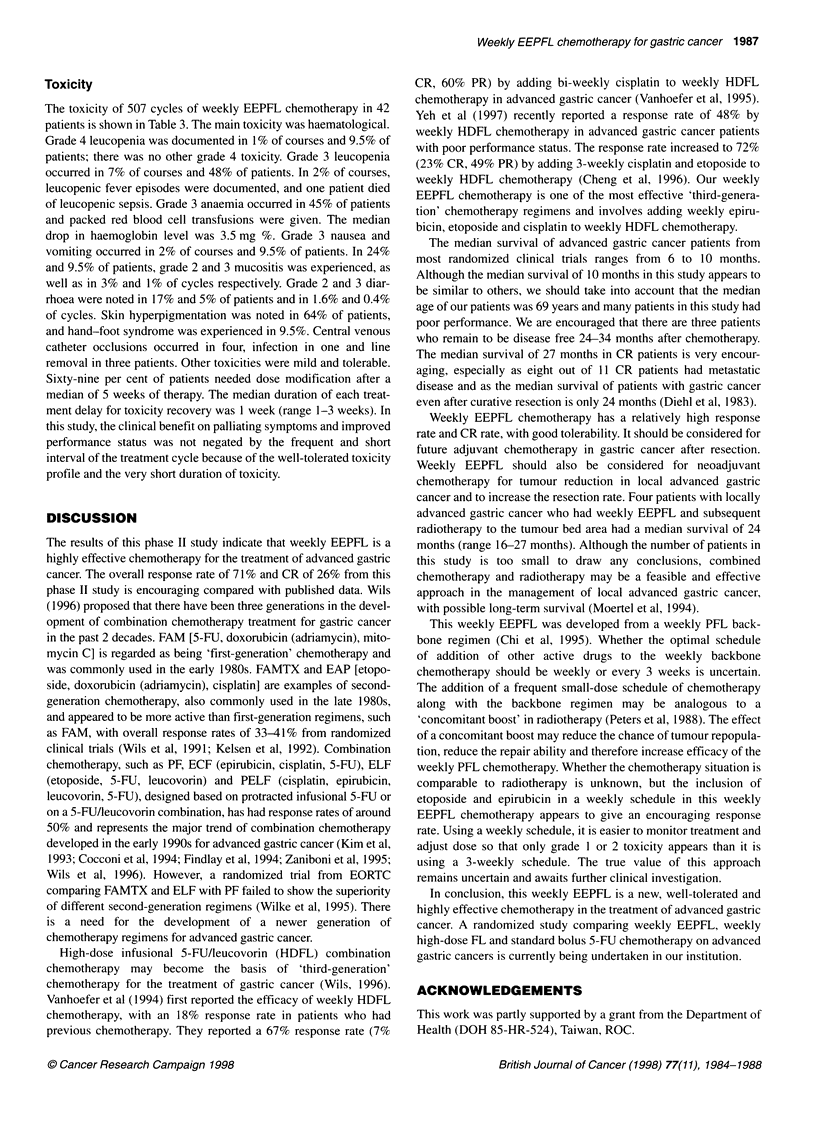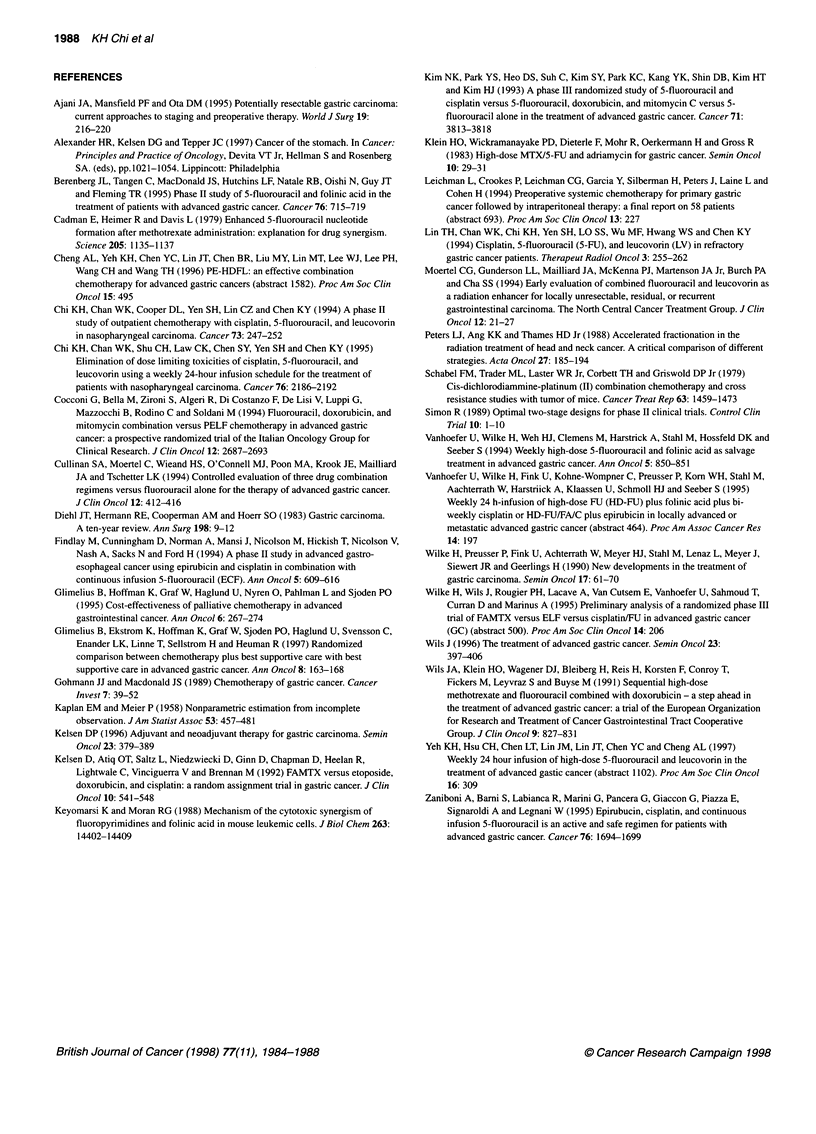# Weekly etoposide, epirubicin, cisplatin, 5-fluorouracil and leucovorin: an effective chemotherapy in advanced gastric cancer.

**DOI:** 10.1038/bjc.1998.329

**Published:** 1998-06

**Authors:** K. H. Chi, Y. Chao, W. K. Chan, S. S. Lo, S. Y. Chen, S. H. Yen, K. Y. Chen, C. W. Wu, S. D. Lee, W. Y. Lui

**Affiliations:** Cancer Center, Veterans General Hospital-Taipei, National Yang-Ming University, Taiwan.

## Abstract

In order to optimize the therapeutic index of combining etoposide, epirubicin, cisplatin, 5-fluorouracil (5-FU), leucovorin (EEPFL) chemotherapy in the treatment of advanced gastric cancer, a trial of a novel schedule of weekly administration was conducted. Weekly EEPFL treatment consisted of a concomitant boost of etoposide 40 mg m(-2) i.v. over 30 min, epirubicin 10 mg m(-2) i.v. over 5 min to a backbone regimen, weekly PFL chemotherapy with cisplatin 25 mg m(-2), 5-FU 2200 mg m(-2), leucovorin 120 mg m(-2) given simultaneously by 24-h i.v. infusion. Response, survival and toxicity were evaluated. Forty-two patients were studied. Median age was 69 (range 31-84) years. Twenty-six per cent of patients showed complete response and 45% partial response. The overall response rate was 71% (95% confidence interval 58-84%). For a total of 507 weekly EEPFL cycles delivered, the incidence of grade 4 leucopenia was 1% of cycles. One patient died of neutropenia septicaemia. There was no other grade 4 toxicity. Grade 3 and 2 leucopenia occurred in 7% and 14% of cycles. The incidence of grade 3 and 2 mucositis was 1% and 3% of cycles. Grade 3 and 2 diarrhoea occurred in 0.4% and 1.6% of cycles. Overall median survival was 10 months (range 3-41+ months). Weekly EEPFL chemotherapy is an effective regimen with tolerable toxicities in the treatment of advanced gastric cancer. A randomized controlled clinical trial to formally assess the efficacy and benefit of EEPFL chemotherapy is under way.


British Journal of Cancer (1998) 77(11), 1984–1988
© 1998 Cancer Research Campaign

### Weekly etoposide, epirubicin, cisplatin, 5-fluorouracil and leucovorin: an effective chemotherapy in advanced gastric cancer

**KH Chi[1,2], Y Chao[3], WK Chan[1], SS Lo[4], SY Chen[1], SH Yen[1], KY Chen[1], CW Wu[4], SD Lee[3] and WY Lui[4]**

[1]Cancer Center, Veterans General Hospital-Taipei, National Yang-Ming University, Taiwan; [2]Associated Faculty, Kaohsiung Medical College, Taiwan; [3]Department of Internal Medicine, [4]Department of Surgery, Veterans General Hospital-Taipei, National Yang-Ming University, Taiwan

**Summary** In order to optimize the therapeutic index of combining etoposide, epirubicin, cisplatin, 5-fluorouracil (5-FU), leucovorin (EEPFL) chemotherapy in the treatment of advanced gastric cancer, a trial of a novel schedule of weekly administration was conducted. Weekly EEPFL treatment consisted of a concomitant boost of etoposide 40 mg $m^{-2}$ i.v. over 30 min, epirubicin 10 mg $m^{-2}$ i.v. over 5 min to a backbone regimen, weekly PFL chemotherapy with cisplatin 25 mg $m^{-2}$, 5-FU 2200 mg $m^{-2}$, leucovorin 120 mg $m^{-2}$ given simultaneously by 24-h i.v. infusion. Response, survival and toxicity were evaluated. Forty-two patients were studied. Median age was 69 (range 31–84) years. Twenty-six per cent of patients showed complete response and 45% partial response. The overall response rate was 71% (95% confidence interval 58–84%). For a total of 507 weekly EEPFL cycles delivered, the incidence of grade 4 leucopenia was 1% of cycles. One patient died of neutropenia septicaemia. There was no other grade 4 toxicity. Grade 3 and 2 leucopenia occurred in 7% and 14% of cycles. The incidence of grade 3 and 2 mucositis was 1% and 3% of cycles. Grade 3 and 2 diarrhoea occurred in 0.4% and 1.6% of cycles. Overall median survival was 10 months (range 3–41+ months). Weekly EEPFL chemotherapy is an effective regimen with tolerable toxicities in the treatment of advanced gastric cancer. A randomized controlled clinical trial to formally assess the efficacy and benefit of EEPFL chemotherapy is under way.

**Keywords:** gastric cancer; weekly administration; etoposide; epirubicin; cisplatin; 5-fluorouracil; leucovorin; chemotherapy

Gastric cancer is one of the most common cancers in the world. Only 40% of patients are amenable to potentially curative surgery (Ajani et al, 1995). Post-operative gastric cancer adjuvant strategies have not yet been decisively successful in improving overall survival (Kelsen, 1996). Gastric cancer is the most chemosensitive cancer of the gastrointestinal tract. Response rates from 8% to 30% from single-agent chemotherapy have been reported (Alexander et al, 1997). Although higher response rates were reported using combination chemotherapy, randomized controlled clinical studies showed no survival benefits when combination chemotherapy was compared with single agent (Kim et al, 1993; Cullinan et al, 1994). Randomized clinical trials also failed to show survival benefit from various combination chemotherapy regimens (Cocconi et al, 1994; Wilke et al, 1995). Only a few trials have reported results that indicate survival, palliative or cost benefit for patients treated with chemotherapy (Glimelius et al, 1995, 1997). Therefore, new effective and tolerable chemotherapy is urgently needed for the treatment of advanced gastric cancer patients.

5-Fluorouracil (5-FU) represents the most extensively studied single agent. Various schedules and dose intensities of 5-FU have been studied, and response rates of about 20% have been reported (Gohmann et al, 1989). Various methods to potentiate 5-FU anticancer activity have been tried in gastric cancer patients. Biochemical modulation of 5-FU (F) by methotrexate or leucovorin (L) has been found to enhance 5-FU-induced cytotoxicity (Cadman et al, 1979; Keyomarsi and Morar, 1988). The combination of 5-FU, doxorubicin (adriamycin), methotrexate (FAMTX) and FL has resulted in 29–59% response rates (Klein et al, 1983; Berenberg et al, 1995). Cisplatin and 5-FU (PF) are synergistic in vitro (Schabel et al, 1979), and PF chemotherapy has resulted in a 51% response in advanced gastric cancer patients (Kim et al, 1993). Etoposide and epirubicin are regarded as being active agents for the treatment of gastric cancers and have been integrated into many combination chemotherapy regimens (Wilke et al, 1990; Findlay et al, 1994; Zaniboni et al, 1995). The combination of cisplatin and a 21-day infusion of 5-FU and leucovorin (PFL) has produced a 48% response rate (Leichman et al, 1994). We found that 96-h infusional PFL chemotherapy every 3 weeks also produced anti-cancer activity in refractory gastric cancer, with a 27% response rate (Lin et al, 1994).

In an attempt to further potentiate the therapeutic effect of PFL chemotherapy in gastric cancer, we added two more active drugs, etoposide and epirubicin, into our 3-weekly PFL chemotherapy regimen (Chi et al, 1994). A pilot study of combining etoposide, epirubicin, cisplatin, 5-FU and leucovorin chemotherapy (EEPFL) given every 3 weeks was performed. Encouraging anti-cancer activity but excessive grade 4 neutropenia and grade 3 mucositis were observed. We then performed a second pilot study of weekly EEPFL chemotherapy in gastric cancer using the same drugs and dose intensity of E, E, P and L but changing the chemotherapy schedule from 96 h infusion every 3 weeks to 24 h infusion weekly, based on our previous experience that PFL chemotherapy dose-limiting toxicity can be eliminated, anti-cancer effect maintained

*Received 9 May 1997*
*Revised 11 September 1997*
*Accepted 1 October 1997*

*Correspondence to:* W-Y Lui, Department of Surgery, Veterans General Hospital-Taipei, # 201, Sec. 2, Shih-Pai Road, Taipei 11217, Taiwan and the dose intensity of 5-FU doubled by using a weekly 24-h PFL infusional schedule (Chi et al, 1995). The results of our second pilot study indicated that changing schedule from every 3 weeks to weekly may result in a high therapeutic index. We therefore performed this phase II clinical trial to further investigate the efficacy of weekly EEPFL chemotherapy in the treatment of advanced gastric cancer patients.

### PATIENTS AND METHODS

### Patient selection

Patients with histologically proven gastric adenocarcinoma with primarily unresectable, locally recurrent after gastrectomy or metastatic diseases were eligible. All patients had no prior chemotherapy. Bidimensionally measurable disease was mandatory even for patients with locally advanced disease. Eastern Cooperative Oncology Group (ECOG) performance status (PS) of 3 or better, adequate bone marrow function (leucocyte count > 4000 $ul^{-1}$, platelet count > 100 000 $ul^{-1}$), renal function (serum creatinine < 1.6 mg $dl^{-1}$), cardiac function (ejection fraction > 45%) and liver function (bilirubin < 2 mg $dl^{-1}$) were required. All patients gave informed consent. The protocols have been carried out with ethical committee approval.

### Treatment plan

Weekly EEPFL treatment consisted of weekly etoposide 40 mg $m^{-2}$ i.v. over 30 min, weekly epirubicin 10 mg $m^{-2}$ i.v. over 5 min and cisplatin 25 mg $m^{-2}$, 5-FU 2200 mg $m^{-2}$, leucovorin 120 mg $m^{-2}$ given simultaneously by weekly 24-h i.v. infusion. (Chi et al, 1994). The chemotherapy can be given as an outpatient regimen with a portable infusion pump (Baxter HealthCare, Deerfield, IL, USA) through implanted central venous access. Dose modifications were based on the degree of leucopenia and thrombocytopenia as determined on day 1 of each weekly cycle immediately before chemotherapy. Full-dose EEPFL chemotherapy would be delivered if WBC was > 2500 $ul^{-1}$ and platelet > 75 000 $ul^{-1}$. Etoposide and epirubicin were omitted for WBC between 2000 and 2500 $ul^{-1}$. Weekly EEPFL would be delayed at least 1 week for WBC < 2000 $ul^{-1}$ or grade 2–4 mucositis or diarrhoea. Patients would be taken off study if treatment delay was more than 3 weeks. Cisplatin was not given for serum creatinine > 2 mg $ml^{-1}$. Etoposide and epirubicin were reduced 25% or 50% for grade 3 or grade 4 neutropenia nadir during chemotherapy. 5-FU was reduced 10% or 20% for grade 2 or 3 mucositis/diarrhoea during chemotherapy. Weekly EEPFL chemotherapy would be discontinued when disease progression, intolerance to therapy or a stable disease after an 8-weekly cycle of therapy was encountered. Generally, another 6-weekly cycle of therapy would be given whenever a complete or partial response was first achieved.

### Response and toxicity criteria

Evaluation procedures, including physical examination, gastroscopy and biopsy, complete blood count, blood chemistry, tumour markers (CEA, CA-199, CA-125), chest radiography, computerized tomography scan of the abdomen and sonography of the abdomen, were performed before chemotherapy. Complete blood counts were performed weekly and blood chemistry every 3 weeks. Physical measurable tumour and treatment toxicity were evaluated weekly before each treatment. Tumour markers, organ imaging and other radiological studies were performed every 1 or 2 months. Complete response (CR) was defined as disappearance of all clinical evidence of disease with normalization of tumour markers. Partial response (PR) was defined as diminution of ≥50% reduction in the sum of products of the perpendicular diameters of all the initial measurable masses for at least 4 weeks. Progressive disease (PD) was defined as a 25% increase in the products of measurable diameters, or the development of a new lesion. Stable disease (SD) was any measurement not fulfilling the criteria for response or progression.

**Table 1** Patient characteristics

| | |
|---|---|
| Age (years) | |
| Median (range) | 69 (31–84) |
| Sex | |
| Male/female | 37/5 |
| Performance status | |
| 0–1 | 23 |
| 2 | 11 |
| 3 | 8 |
| Post-gastrectomy | |
| Locally recurrent | 3 |
| Metastatic | 20 |
| Non-resectable | |
| Locally advanced | 9 |
| Metastatic | 10 |
| Sites of disease | |
| Stomach | 12 |
| Liver | 19 |
| Intra-abdominal lymph node | 13 |
| Supraclavicular lymph node | 3 |
| Lung | 4 |
| Pelvic mass | 4 |
| Ascites | 3 |
| Skin | 1 |
| Tumour markers | 34 |

**Table 2** Response

| | Patients | % |
|---|---|---|
| CR | 11 | 26 |
| PR | 19 | 45 |
| SD | 6 | 14 |
| PD | 4 | 10 |
| Not evaluable | 2 | 5 |
| Total | 42 | 100 |

### Statistical methods

The Simon two-stage phase II clinical trial design was used (Simon, 1989), with 23 patients in the first stage and 39 patients in the second stage. If the response rate was less than 11 out of 23 patients in the first stage or 23 out of 39 patients in the second stage, the treatment would be rejected, with a response rate of 50% or 70%, respectively, with $\alpha$ of 0.1 and $\beta$ of 0.1. Response rates were compared using Fisher's exact test. Survival was calculated using the Kaplan and Meier method (Kaplan and Meier, 1958). The time to progression was measured from the time of first-study drug administration to documented progressive disease.

© Cancer Research Campaign 1998

### RESULTS

Between August 1992 and November 1995, 42 patients were studied. Forty patients were evaluable for response. One patient was lost to follow-up after two cycles of therapy. One patient had inadequate infusion time for the first cycle of therapy and did not return for the second cycle of therapy. Patient characteristics are listed in Table 1. There were 37 male and five female patients. The median age was 69 years. There were 34 patients with ECOG PS 0–2 and eight patients with grade 3 PS. Nineteen patients were inoperable because of locally advanced disease or metastatic disease. Twenty-three patients had previous gastrectomy for gastric cancer and presented with local recurrence or metastasis. No patient had previous chemotherapy.

### Response and survival

The median cycles of weekly EEPFL chemotherapy given was 12 (range 1–30). The response rates are summarized in Table 2. There were 26% CR and 45% PR observed. The overall response rate (CR + PR) was 71% (confidence interval 58–84%). Three clinical CR patients (one local disease and two metastasis) had laparotomy after weekly EEPFL chemotherapy, and all had pathological CR. There were 14% SD and 10% PD. The overall response rate was 79% in patients with ECOG PS 0–2 and 37.5% in patients with ECOG PS 3, this being statistically significant ($P = 0.03$). There was no statistically significant difference in response rates between patients with local disease vs metastasis, gastrectomy vs no gastrectomy, young vs old age (≥ 60 years), sites of metastasis, < 3 vs ≥ 3 tumour sites or ascites.

Relief from disease-related symptoms developed gradually, but significantly, if response to chemotherapy was achieved; in particular, relief from various pain and epigastric fullness sensations was noted. The performance status was improved in 53% of patients who had original PS of 2–3 and maintained in 83% of patients who had original PS of 0–1. The median time to CR was 12 weeks (range 8–16 weeks). The median time to PR was 8 weeks (range 3–16 weeks). The median response duration of CR was 15 months (range 6–34 months). The median duration of PR was 5 months (range 2–18 months). The survival rates for all and different groups of patients treated with weekly EEPFL chemotherapy are shown in Figures 1 and 2. The overall median survival was 10 months (range 3–41 months). The median survival of the CR patients was 27 months (range 6–41+ months). The median survival of PR patients was 15 months (range 3–24 months). The median survival times for patients with localized disease and metastatic disease were 18 and 9.5 months respectively ($P = 0.9$). The median progression-free survival times for patients with localized or metastatic diseases were both 5 months. Three patients in this study remained disease free and alive at 24+, 26+ and 34+ months.

**Figure 1** Survival of patients treated with the weekly EEPFL chemotherapy

**Figure 2** Survival of localized or metastatic gastric cancer patients treated with weekly EEPFL chemotherapy

**Table 3** Percentage toxicity from weekly EEPFL chemotherapy

| | Per cycle analysis (*n* = 507) Grade (%) | | | | Per patient analysis (*n* = 42) Grade (%) | | | |
|---|---|---|---|---|---|---|---|---|
| **Toxicity** | **1** | **2** | **3** | **4** | **1** | **2** | **3** | **4** |
| Leucopenia | 18 | 14 | 7 | 1 | 12 | 24 | 48 | 9.5 |
| Thrombocytopenia | 6 | 3 | 1 | 0 | 17 | 9.5 | 7 | 0 |
| Anaemia | 35 | 30 | 5 | 0 | 7 | 36 | 45 | 0 |
| Nausea/vomiting | 53 | 10 | 2 | 0 | 48 | 12 | 9.5 | 0 |
| Mucositis | 8 | 3 | 1 | 0 | 36 | 24 | 9.5 | 0 |
| Diarrhoea | 2 | 1.6 | 0.4 | 0 | 24 | 17 | 5 | 0 |
| Nephrotoxicity | 0.6 | 0.4 | 0 | 0 | 7 | 5 | 0 | 0 |
| Neurotoxicity | 1 | 0 | 0 | 0 | 5 | 0 | 0 | 0 |

 © Cancer Research Campaign 1998

### Toxicity

The toxicity of 507 cycles of weekly EEPFL chemotherapy in 42 patients is shown in Table 3. The main toxicity was haematological. Grade 4 leucopenia was documented in 1% of courses and 9.5% of patients; there was no other grade 4 toxicity. Grade 3 leucopenia occurred in 7% of courses and 48% of patients. In 2% of courses, leucopenic fever episodes were documented, and one patient died of leucopenic sepsis. Grade 3 anaemia occurred in 45% of patients and packed red blood cell transfusions were given. The median drop in haemoglobin level was 3.5 mg %. Grade 3 nausea and vomiting occurred in 2% of courses and 9.5% of patients. In 24% and 9.5% of patients, grade 2 and 3 mucositis was experienced, as well as in 3% and 1% of cycles respectively. Grade 2 and 3 diarrhoea were noted in 17% and 5% of patients and in 1.6% and 0.4% of cycles. Skin hyperpigmentation was noted in 64% of patients, and hand–foot syndrome was experienced in 9.5%. Central venous catheter occlusions occurred in four, infection in one and line removal in three patients. Other toxicities were mild and tolerable. Sixty-nine per cent of patients needed dose modification after a median of 5 weeks of therapy. The median duration of each treatment delay for toxicity recovery was 1 week (range 1–3 weeks). In this study, the clinical benefit on palliating symptoms and improved performance status was not negated by the frequent and short interval of the treatment cycle because of the well-tolerated toxicity profile and the very short duration of toxicity.

### DISCUSSION

The results of this phase II study indicate that weekly EEPFL is a highly effective chemotherapy for the treatment of advanced gastric cancer. The overall response rate of 71% and CR of 26% from this phase II study is encouraging compared with published data. Wils (1996) proposed that there have been three generations in the development of combination chemotherapy treatment for gastric cancer in the past 2 decades. FAM [5-FU, doxorubicin (adriamycin), mitomycin C] is regarded as being 'first-generation' chemotherapy and was commonly used in the early 1980s. FAMTX and EAP [etoposide, doxorubicin (adriamycin), cisplatin] are examples of second-generation chemotherapy, also commonly used in the late 1980s, and appeared to be more active than first-generation regimens, such as FAM, with overall response rates of 33–41% from randomized clinical trials (Wils et al, 1991; Kelsen et al, 1992). Combination chemotherapy, such as PF, ECF (epirubicin, cisplatin, 5-FU), ELF (etoposide, 5-FU, leucovorin) and PELF (cisplatin, epirubicin, leucovorin, 5-FU), designed based on protracted infusional 5-FU or on a 5-FU/leucovorin combination, has had response rates of around 50% and represents the major trend of combination chemotherapy developed in the early 1990s for advanced gastric cancer (Kim et al, 1993; Cocconi et al, 1994; Findlay et al, 1994; Zaniboni et al, 1995; Wils et al, 1996). However, a randomized trial from EORTC comparing FAMTX and ELF with PF failed to show the superiority of different second-generation regimens (Wilke et al, 1995). There is a need for the development of a newer generation of chemotherapy regimens for advanced gastric cancer.

High-dose infusional 5-FU/leucovorin (HDFL) combination chemotherapy may become the basis of 'third-generation' chemotherapy for the treatment of gastric cancer (Wils, 1996). Vanhoefer et al (1994) first reported the efficacy of weekly HDFL chemotherapy, with an 18% response rate in patients who had previous chemotherapy. They reported a 67% response rate (7% CR, 60% PR) by adding bi-weekly cisplatin to weekly HDFL chemotherapy in advanced gastric cancer (Vanhoefer et al, 1995). Yeh et al (1997) recently reported a response rate of 48% by weekly HDFL chemotherapy in advanced gastric cancer patients with poor performance status. The response rate increased to 72% (23% CR, 49% PR) by adding 3-weekly cisplatin and etoposide to weekly HDFL chemotherapy (Cheng et al, 1996). Our weekly EEPFL chemotherapy is one of the most effective 'third-generation' chemotherapy regimens and involves adding weekly epirubicin, etoposide and cisplatin to weekly HDFL chemotherapy.

The median survival of advanced gastric cancer patients from most randomized clinical trials ranges from 6 to 10 months. Although the median survival of 10 months in this study appears to be similar to others, we should take into account that the median age of our patients was 69 years and many patients in this study had poor performance. We are encouraged that there are three patients who remain to be disease free 24–34 months after chemotherapy. The median survival of 27 months in CR patients is very encouraging, especially as eight out of 11 CR patients had metastatic disease and as the median survival of patients with gastric cancer even after curative resection is only 24 months (Diehl et al, 1983).

Weekly EEPFL chemotherapy has a relatively high response rate and CR rate, with good tolerability. It should be considered for future adjuvant chemotherapy in gastric cancer after resection. Weekly EEPFL should also be considered for neoadjuvant chemotherapy for tumour reduction in local advanced gastric cancer and to increase the resection rate. Four patients with locally advanced gastric cancer who had weekly EEPFL and subsequent radiotherapy to the tumour bed area had a median survival of 24 months (range 16–27 months). Although the number of patients in this study is too small to draw any conclusions, combined chemotherapy and radiotherapy may be a feasible and effective approach in the management of local advanced gastric cancer, with possible long-term survival (Moertel et al, 1994).

This weekly EEPFL was developed from a weekly PFL backbone regimen (Chi et al, 1995). Whether the optimal schedule of addition of other active drugs to the weekly backbone chemotherapy should be weekly or every 3 weeks is uncertain. The addition of a frequent small-dose schedule of chemotherapy along with the backbone regimen may be analogous to a 'concomitant boost' in radiotherapy (Peters et al, 1988). The effect of a concomitant boost may reduce the chance of tumour repopulation, reduce the repair ability and therefore increase efficacy of the weekly PFL chemotherapy. Whether the chemotherapy situation is comparable to radiotherapy is unknown, but the inclusion of etoposide and epirubicin in a weekly schedule in this weekly EEPFL chemotherapy appears to give an encouraging response rate. Using a weekly schedule, it is easier to monitor treatment and adjust dose so that only grade 1 or 2 toxicity appears than it is using a 3-weekly schedule. The true value of this approach remains uncertain and awaits further clinical investigation.

In conclusion, this weekly EEPFL is a new, well-tolerated and highly effective chemotherapy in the treatment of advanced gastric cancer. A randomized study comparing weekly EEPFL, weekly high-dose FL and standard bolus 5-FU chemotherapy on advanced gastric cancers is currently being undertaken in our institution.

### ACKNOWLEDGEMENTS

This work was partly supported by a grant from the Department of Health (DOH 85-HR-524), Taiwan, ROC.

© Cancer Research Campaign 1998

### REFERENCES

Ajani JA, Mansfield PF and Ota DM (1995) Potentially resectable gastric carcinoma: current approaches to staging and preoperative therapy. *World J Surg* **19**: 216–220

Alexander HR, Kelsen DG and Tepper JC (1997) Cancer of the stomach. In *Cancer: Principles and Practice of Oncology*, Devita VT Jr, Hellman S and Rosenberg SA. (eds), pp.1021–1054. Lippincott: Philadelphia

Berenberg JL, Tangen C, MacDonald JS, Hutchins LF, Natale RB, Oishi N, Guy JT and Fleming TR (1995) Phase II study of 5-fluorouracil and folinic acid in the treatment of patients with advanced gastric cancer. *Cancer* **76**: 715–719

Cadman E, Heimer R and Davis L (1979) Enhanced 5-fluorouracil nucleotide formation after methotrexate administration: explanation for drug synergism. *Science* **205**: 1135–1137

Cheng AL, Yeh KH, Chen YC, Lin JT, Chen BR, Liu MY, Lin MT, Lee WJ, Lee PH, Wang CH and Wang TH (1996) PE-HDFL: an effective combination chemotherapy for advanced gastric cancers (abstract 1582). *Proc Am Soc Clin Oncol* **15**: 495

Chi KH, Chan WK, Cooper DL, Yen SH, Lin CZ and Chen KY (1994) A phase II study of outpatient chemotherapy with cisplatin, 5-fluorouracil, and leucovorin in nasopharyngeal carcinoma. *Cancer* **73**: 247–252

Chi KH, Chan WK, Shu CH, Law CK, Chen SY, Yen SH and Chen KY (1995) Elimination of dose limiting toxicities of cisplatin, 5-fluorouracil, and leucovorin using a weekly 24-hour infusion schedule for the treatment of patients with nasopharyngeal carcinoma. *Cancer* **76**: 2186–2192

Cocconi G, Bella M, Zironi S, Algeri R, Di Costanzo F, De Lisi V, Luppi G, Mazzocchi B, Rodino C and Soldani M (1994) Fluorouracil, doxorubicin, and mitomycin combination versus PELF chemotherapy in advanced gastric cancer: a prospective randomized trial of the Italian Oncology Group for Clinical Research. *J Clin Oncol* **12**: 2687–2693

Cullinan SA, Moertel C, Wieand HS, O'Connell MJ, Poon MA, Krook JE, Mailliard JA and Tschetter LK (1994) Controlled evaluation of three drug combination regimens versus fluorouracil alone for the therapy of advanced gastric cancer. *J Clin Oncol* **12**: 412–416

Diehl JT, Hermann RE, Cooperman AM and Hoerr SO (1983) Gastric carcinoma. A ten-year review. *Ann Surg* **198**: 9–12

Findlay M, Cunningham D, Norman A, Mansi J, Nicolson M, Hickish T, Nicolson V, Nash A, Sacks N and Ford H (1994) A phase II study in advanced gastro-esophageal cancer using epirubicin and cisplatin in combination with continuous infusion 5-fluorouracil (ECF). *Ann Oncol* **5**: 609–616

Glimelius B, Hoffman K, Graf W, Haglund U, Nyren O, Pahlman L and Sjoden PO (1995) Cost-effectiveness of palliative chemotherapy in advanced gastrointestinal cancer. *Ann Oncol* **6**: 267–274

Glimelius B, Ekstrom K, Hoffman K, Graf W, Sjoden PO, Haglund U, Svensson C, Enander LK, Linne T, Sellstrom H and Heuman R (1997) Randomized comparison between chemotherapy plus best supportive care with best supportive care in advanced gastric cancer. *Ann Oncol* **8**: 163–168

Gohmann JJ and Macdonald JS (1989) Chemotherapy of gastric cancer. *Cancer Invest* **7**: 39–52

Kaplan EM and Meier P (1958) Nonparametric estimation from incomplete observation. *J Am Statist Assoc* **53**: 457–481

Kelsen DP (1996) Adjuvant and neoadjuvant therapy for gastric carcinoma. *Semin Oncol* **23**: 379–389

Kelsen D, Atiq OT, Saltz L, Niedzwiecki D, Ginn D, Chapman D, Heelan R, Lightwale C, Vinciguerra V and Brennan M (1992) FAMTX versus etoposide, doxorubicin, and cisplatin: a random assignment trial in gastric cancer. *J Clin Oncol* **10**: 541–548

Keyomarsi K and Moran RG (1988) Mechanism of the cytotoxic synergism of fluoropyrimidines and folinic acid in mouse leukemic cells. *J Biol Chem* **263**: 14402–14409

Kim NK, Park YS, Heo DS, Suh C, Kim SY, Park KC, Kang YK, Shin DB, Kim HT and Kim HJ (1993) A phase III randomized study of 5-fluorouracil and cisplatin versus 5-fluorouracil, doxorubicin, and mitomycin C versus 5-fluorouracil alone in the treatment of advanced gastric cancer. *Cancer* **71**: 3813–3818

Klein HO, Wickramanayake PD, Dieterle F, Mohr R, Oerkermann H and Gross R (1983) High-dose MTX/5-FU and adriamycin for gastric cancer. *Semin Oncol* **10**: 29–31

Leichman L, Crookes P, Leichman CG, Garcia Y, Silberman H, Peters J, Laine L and Cohen H (1994) Preoperative systemic chemotherapy for primary gastric cancer followed by intraperitoneal therapy: a final report on 58 patients (abstract 693). *Proc Am Soc Clin Oncol* **13**: 227

Lin TH, Chan WK, Chi KH, Yen SH, LO SS, Wu MF, Hwang WS and Chen KY (1994) Cisplatin, 5-fluorouracil (5-FU), and leucovorin (LV) in refractory gastric cancer patients. *Therapeut Radiol Oncol* **3**: 255–262

Moertel CG, Gunderson LL, Mailliard JA, McKenna PJ, Martenson JA Jr, Burch PA and Cha SS (1994) Early evaluation of combined fluorouracil and leucovorin as a radiation enhancer for locally unresectable, residual, or recurrent gastrointestinal carcinoma. The North Central Cancer Treatment Group. *J Clin Oncol* **12**: 21–27

Peters LJ, Ang KK and Thames HD Jr (1988) Accelerated fractionation in the radiation treatment of head and neck cancer. A critical comparison of different strategies. *Acta Oncol* **27**: 185–194

Schabel FM, Trader ML, Laster WR Jr, Corbett TH and Griswold DP Jr (1979) Cis-dichlorodiammine-platinum (II) combination chemotherapy and cross resistance studies with tumor of mice. *Cancer Treat Rep* **63**: 1459–1473

Simon R (1989) Optimal two-stage designs for phase II clinical trials. *Control Clin Trial* **10**: 1–10

Vanhoefer U, Wilke H, Weh HJ, Clemens M, Harstrick A, Stahl M, Hossfeld DK and Seeber S (1994) Weekly high-dose 5-fluorouracil and folinic acid as salvage treatment in advanced gastric cancer. *Ann Oncol* **5**: 850–851

Vanhoefer U, Wilke H, Fink U, Kohne-Wompner C, Preusser P, Korn WH, Stahl M, Aachterrath W, Harstriick A, Klaassen U, Schmoll HJ and Seeber S (1995) Weekly 24 h-infusion of high-dose FU (HD-FU) plus folinic acid plus bi-weekly cisplatin or HD-FU/FA/C plus epirubicin in locally advanced or metastatic advanced gastric cancer (abstract 464). *Proc Am Assoc Cancer Res* **14**: 197

Wilke H, Preusser P, Fink U, Achterrath W, Meyer HJ, Stahl M, Lenaz L, Meyer J, Siewert JR and Geerlings H (1990) New developments in the treatment of gastric carcinoma. *Semin Oncol* **17**: 61–70

Wilke H, Wils J, Rougier PH, Lacave A, Van Cutsem E, Vanhoefer U, Sahmoud T, Curran D and Marinus A (1995) Preliminary analysis of a randomized phase III trial of FAMTX versus ELF versus cisplatin/FU in advanced gastric cancer (GC) (abstract 500). *Proc Am Soc Clin Oncol* **14**: 206

Wils J (1996) The treatment of advanced gastric cancer. *Semin Oncol* **23**: 397–406

Wils JA, Klein HO, Wagener DJ, Bleiberg H, Reis H, Korsten F, Conroy T, Fickers M, Leyvraz S and Buyse M (1991) Sequential high-dose methotrexate and fluorouracil combined with doxorubicin – a step ahead in the treatment of advanced gastric cancer: a trial of the European Organization for Research and Treatment of Cancer Gastrointestinal Tract Cooperative Group. *J Clin Oncol* **9**: 827–831

Yeh KH, Hsu CH, Chen LT, Lin JM, Lin JT, Chen YC and Cheng AL (1997) Weekly 24 hour infusion of high-dose 5-fluorouracil and leucovorin in the treatment of advanced gastic cancer (abstract 1102). *Proc Am Soc Clin Oncol* **16**: 309

Zaniboni A, Barni S, Labianca R, Marini G, Pancera G, Giaccon G, Piazza E, Signaroldi A and Legnani W (1995) Epirubucin, cisplatin, and continuous infusion 5-fluorouracil is an active and safe regimen for patients with advanced gastric cancer. *Cancer* **76**: 1694–1699

*© Cancer Research Campaign 1998*